# Bioaccumulation and Mass Balance Analysis of Veterinary Antibiotics in an Agricultural Environment

**DOI:** 10.3390/toxics10050213

**Published:** 2022-04-24

**Authors:** Jin-Wook Kim, Young-Kyu Hong, Jae-E. Yang, Oh-Kyung Kwon, Sung-Chul Kim

**Affiliations:** 1 Department of Bio-Environmental Chemistry, Chungnam National University, Daejeon 34134, Korea; kin1888@cnu.ac.kr (J.-W.K.); hyk8895@cnu.ac.kr (Y.-K.H.); 2 Department of Biological Environment, Kangwon National University, Chuncheon 24341, Korea; yangjay@kangwon.ac.kr; 3 Biogas Research Center, Hankyung National University, Anseong-si 17579, Korea

**Keywords:** crop, fate, manure, mass balance, soil, veterinary antibiotics

## Abstract

Veterinary antibiotics (VAs) released into the environment are a concern because of the possibility for increasing antibiotic-resistance genes. The concentrations of six VAs, chlortetracycline, oxytetracycline, tetracycline, sulfamethazine, sulfamethoxazole, and sulfathiazole, in manure-based compost, soil, and crops were measured using liquid chromatography–tandem mass spectrometry. Mass balance analysis was conducted based on the measured antibiotic concentration, cultivation area, and amount of manure-based compost applied. The result showed that the detected mean concentration of VAs ranges was 3.52~234.19 μg/kg, 0.52~13.08 μg/kg, and 1.05~39.57 μg/kg in manure-based compost, soil, and crops, respectively, and the substance of VAs detected in different media was also varied. Mass balance analysis showed that the VAs released from the manure-based compost can remain in soil (at rates of 26% to 100%), be taken up by crops (at rates of 0.4% to 3.7%), or dissipated (at rates of 9% to 73%) during the cultivation period. Among the six VAs, chlortetracycline and oxytetracycline mainly remained in the soil, whereas sulfamethoxazole and sulfathiazole were mainly dissipated. Although we did not verify the exact mechanism of the fate and distribution of VAs in this study, our results showed that these can vary depending on the different characteristics of VAs and the soil properties.

## 1. Introduction

Veterinary antibiotics (VAs) have been used for therapeutic purposes to treat and prevent diseases caused by pathogenic bacteria, or for nontherapeutic purposes to promote the growth of livestock [1]. According to a previous report [2], global antibiotic use is expected to increase by 67% by 2030, and the increased usage of VAs could have a detrimental effect on the ecosystem [3]. In general, 10–20% of VAs administered to livestock are metabolized in the animal’s body, and the remaining 80–90% are excreted in urine and manure [4]. Livestock manure and urine are generally utilized as manure-based compost or liquid fertilizer and applied to soil for improving soil quality and supplying nutrients to crops in agricultural environments [5]. This indicates that residuals of VAs remaining in manure-based compost or liquid fertilizer can be retained in the soil [6] or transferred to other environmental compartments via leaching into groundwater or runoff to surface water [7,8]. VAs retained in the soil can also be accumulated in different parts of the crops, causing growth inhibition [9,10,11]. In addition, released VAs can reduce the diversity and activity of soil bacteria [12,13], as well as increasing antibiotic-resistant genes (ARGs) in the agricultural environment [14,15].

Several countries, including the United States [16], European nations [17], Canada [18], and China [19], have reported the occurrence of VAs in agricultural environments. The highest concentrations of VAs in the different compartments of manure, soil, and crops were reportedly up to 143.97 mg/kg, 1.59 mg/kg, and 0.53 mg/kg, respectively. However, many studies have focused on monitoring VAs in a single medium, and their distribution in multiple agricultural environments, such as combinations of manure-based compost, soil, and crop systems, is not fully understood. Mass balance analysis can be an effective method to understand the fate and distribution in multiple media by comparing the relative mass of VAs remaining in each medium [20]. A prior mass balance study showed that most of the released antibiotics from manure remained in soil (about 65%), and less than 0.1% of the antibiotics were accumulated in the plants [20]. This study clearly showed that the fate of different antibiotics varied depending on the different properties of antibiotics and uptake mechanisms of crops.

The fate of antibiotics is highly affected by soil properties such as soil pH, organic carbon contents, and cation exchange capacity (CEC) [21]. Antibiotics are generally polar and can be ionizable depending on pK_a_ values and soil pH, resulting in cationic, anionic, zwitterionic, and neutral species [22]. Park and Huwe (2022) evaluated the transport of sulfonamide in agricultural soil and reported that the adsorption affinity of sulfonamide was highest in the pH range of 4.0–8.0 [23]. The organic carbon contents also play an important role in controlling the fate and transport of antibiotics, exerting hydrophobic interactions. In addition, higher clay contents can increase the sorption of antibiotics because of their large specific surface area and ion exchange capacity [21]. 

The objective of this study was to investigate the fate and distribution of VAs, including tetracycline (TC) and sulfonamide (SA) antibiotics, in agricultural environments. Antibiotics were analyzed in manure-based compost, soil, and crop samples collected during the crop-growing period, and mass balance analysis was conducted to examine their fate and distribution in the manure-based compost–soil–crop system.

## 2. Materials and Methods

### 2.1. Chemicals and Standards

Veterinary antibiotics were selected based on their sales in Korea, and included chlortetracycline (CTC), oxytetracycline (OTC), tetracycline (TC), sulfamethazine (SMZ), sulfamethoxazole (SMX), and sulfathiazole (STZ). All solvents used for pretreatment and instrumental analyses were of HPLC-grade and purchased from JT Baker (Philipsburg, NJ, USA). Sodium phosphate dibasic, formic acid, and Na_2_-EDTA were obtained from Sigma-Aldrich (St. Louis, MO, USA), and citric acid was purchased from Daejung Chemicals & Metals Co. (Gyeonggi, Korea). Standard solutions of CTC hydrochloride (64-72-2, ≥97.0%), OTC hydrochloride (2058-46-0, ≥94.9%), TC hydrochloride (64-75-5, ≥95%), SMZ (57-68-1, ≥99%), SMX (723-46-6 ≥98%), and SZ (72-14-0, ≥98.0%) were prepared with standards purchased from Sigma-Aldrich (St. Louis, MO, USA). The stock solution (100 mg/L) was prepared by dissolving the antibiotic standard in methanol, and the working solution was prepared by sequentially diluting the stock solution with methanol to an appropriate concentration. In addition, simeton, an internal standard at a concentration of 1000 mg/L, was purchased from AccuStandard (New Haven, CT, USA) and diluted with methanol. Individual standard solutions and internal standards were stored in amber glass bottles at −20 °C. 

### 2.2. Site Description and Sample Collection

Manure-based compost, soil, and crop samples were collected from three sampling sites located in Chungnam and Jeonbuk provinces in Korea. The experimental area of each site was 100 m^2^ (10 m × 10 m, W × L) and 3 different crops—perilla (*perilla frutescens*), maize (*zea mays*), and soybean (*glycine max*)—were cultivated from April to September. The temperature and annual rainfall of the sampling sites during crop cultivation were 6.1–31.3 °C and 1191–1354 mm, respectively (Figure S1 in Supplementary Materials).

Commercially available manure-based compost was purchased from the market at each sampling site. Although the exact mixing ratio between the livestock manure and organic bedding material in each compost was not known, a mixing ratio of 70% livestock manure (swine, cattle, or poultry) and 30% organic bedding materials is common in commercially available manure-based compost in Korea. From each sampling site, manure-based compost was collected before applying it to the soil in March. Approximately 100 g of manure-based compost was collected from 5 different packages and combined in a plastic sample bag to make one representative sample for each sampling site. Then, manure-based compost was applied in the field at an application rate of 1.5 ton/ha, as recommended by the Rural Development Agency (RDA) in Korea.

Soil samples were collected at a depth of 0–20 cm using a hand auger after removing crop residuals and stones on the surface of the soil. Similar to manure-based compost sample collection, five soil samples were collected from the 5 different locations in each sampling site and combined in a plastic sample bag to make one representative soil sample.

Crop samples were collected after harvesting in September. Three pieces of crop samples were collected from 5 locations in each sampling site, separated by edible parts, stems, and roots, and contained separately in a plastic sample bag. All samples were stored in an iced cooler and transferred to a laboratory for analysis.

The soil and manure-based compost samples were oven-dried (JSOF-150, JSR, Tokyo, Japan) at 105 °C for 24 h for physicochemical analyses. For residual VA analysis, air-dried samples at 20 °C under dark conditions were used. Crop samples were freeze-dried using a freeze-dryer (SFDSF12, SAMWON, Seoul, Korea), and finely pulverized with a mortar for VA analysis.

### 2.3. Physicochemical Analysis of Samples

The soil bulk density was measured using 100 cm^3^ stainless-steel soil cores [24], and the soil texture was classified using a hydrometer based on the United States Department of Agriculture (USDA) triangle method. Soil pH and electrical conductivity (EC) were measured at a soil:distilled water ratio of 1:5 (*w*/*v*), or manure-based compost:distilled water ratio of 1:10 (*w*/*v*) using a pH meter (Orion Star™ A111, Thermo Fisher Scientific, Waltham, MA, USA) and an EC meter (SevenCompact Conductivity Meter S230, Mettler Toledo, Columbus, OH, USA), according to the Korean Standard Test Method (KSTM ES 07302). Soil organic matter content (SOM) was analyzed using the Walkley–Black method [25], and the organic content of compost was measured using a loss-on-ignition method with a furnace (Lindberg/Blue M42.5 LC2 Moldatherm Box Furnace, Thermo Fisher Scientific).

### 2.4. Antibiotic Extraction and Clean-Up Process

For antibiotic extraction, 1.0 g of compost and soil, or 0.1 g of crop sample, was accurately weighed in a 50 mL centrifuge tube containing 20 mL of Na_2_EDTA-McIlvain buffer (pH 4.0). The solution was mixed using an orbital shaker for 15 min, centrifuged at 4000× *g* rpm for 15 min, and the supernatant was transferred into 250 mL flasks. The same sample was extracted again; in total, 40 mL of supernatant was combined, diluted to 120 mL with ultrapure water, and filtered using a 0.22 μm cellulose acetate membrane filter. Solid-phase extraction was performed using an Oasis HLB Extraction Cartridge (3 cc/60 mg, Water, Milford, MA, USA) to purify the extracted sample. The cartridge was conditioned with 3 mL of methanol, 3 mL of 0.5 M HCl, and 3 mL of purified water. The sample was loaded into the cartridge at 4 mL/min. After all samples had passed through the cartridge, it was washed with 9 mL of ultrapure water and dried under vacuum for 10 min. Finally, the antibiotics were eluted with 5 mL of methanol into a 15 mL glass centrifuge tube, and 50 μL of 0.24 mg/L simeton, an internal standard, was added. The eluent was evaporated using a nitrogen evaporator (12 Position N-EVAP Nitrogen Evaporator, Organization, Berlin, MA, USA) at 50 °C and reconstituted with 120 μL of mobile phase A (0.1% formic acid in HPLC water). The extract was placed in a 1.5 mL centrifuge tube (spin-x centrifuge tube filter, Corning Incorporated, Corning, NY, USA) containing a 0.22 μm nylon filter and centrifuged at 15,000× *g* rpm for 3 min. Finally, the filtered extract was transferred to a 2 mL amber glass vial and stored at −20 °C in a freezer until further analysis.

### 2.5. Instrumental Analysis

The target antibiotics extracted from all the samples and standard antibiotic solutions were determined and quantified using an Agilent 1290 Infinity II (Agilent, Santa Clara, CA, USA) system coupled to a triple quadrupole mass spectrometer (6500 Qtrap, SCIEX, Framingham, MA, USA) equipped with an electrospray ionization source (ESI); all antibiotics were analyzed using multiple reaction monitoring (MRM) in positive ion electrospray mode. The target antibiotics were separated using a reversed-phase Zorbax Eclipse Plus-C18 column (4.6 × 150 mm, 3.5 µm, Agilent, Santa Clara, CA, USA); mobile phase A (0.1% formic acid in HPLC-grade water) and mobile phase B (0.1% formic acid in acetonitrile) were used for the gradient elution system. Detailed liquid chromatography–tandem mass spectrometry (LC–MS/MS) conditions and MRM parameters are presented in Tables S1 and S2, respectively, in Supplementary Materials.

### 2.6. Method Validation

The analytical method for residual antibiotics was validated in terms of the linearity, accuracy, method detection limit (MDL), and limit of quantification (LOQ), following the guidelines of the U.S. Environmental Protection Agency (USEPA) method 1694 [26]. The calibration curve in the range of 0.01–1.00 mg/kg was generated by linear fit, and the linearity of each antibiotic was evaluated by the coefficient of determination (R^2^). The R^2^ value for all antibiotics was above 0.99, indicating a good linearity for the calibration curves of all target analytes.

The method accuracy was estimated as the percentage recovery (%) of antibiotics from the manure-based compost, soil, and crop samples spiked with standard mixtures. The blank samples containing no residual antibiotics for all three matrices were spiked to a final concentration of 1.0 mg/kg, and antibiotic concentrations were measured following the same procedure as the samples. Commercially available antibiotic-free manure-based compost was used as a blank sample. In addition, blank samples of soil and crop (soybean) were obtained from the upland field where only chemical fertilizer has been applied for 40 years in the Rural Development Agency (RDA), Korea. All blank samples were confirmed to contain no residual VAs after following the same measurement procedure as the samples. Then, the recovery was expressed as a mean percentage of three replicates by comparing the measured concentration with the actual spiked concentration. All measured concentrations of VAs in samples were calculated based on recovery and no surrogate was used for VAs analysis.

The method detection limit (MDL) and limit of quantification (LOQ) were evaluated by analyzing replicate samples spiked at a low concentration level (0.01 mg/kg), using the following formulae (Equations (1) and (2)):

MDL (ng/kg) = t _(n−1, 1−__α__=__0.98)_ × SD (1)

LOQ (ng/kg) = 10 × SD(2)

where t _(n−1, 1−α=0.98)_ is the Student’s t distribution value at the 98% confidence level, n − 1 degrees of freedom, and SD is the standard deviation of replicate spiked samples (n = 7). The LOQ was calculated as 10 times the standard deviation of the spiked sample. The linearity, accuracy, MDL, and LOQ results in manure-based compost, soil, and crops are presented in Table 1.

### 2.7. Mass Balance Analysis

The mass of antibiotics in the manure-based compost, soil, and crops was calculated using Equations (3)–(5). 

The equations are as follows:

M_compost_ = C_compost_ × A × F (3)

M_soil_ = C_soil_ × A × D × BD (4)

M_crop_ = C_crop_ × A × Y (5)

where M_sample_ is the calculated mass of antibiotics in each sample (μg), C_sample_ is the antibiotic concentration in each sample (μg/kg), A is the cultivation area (m^2^), F is the fertilizer application amount (kg/m^3^), BD is the soil bulk density (kg/m^3^), D is the depth (m), and Y is the crop yield (kg/m^2^). 

The initial concentration of antibiotics before crop cultivation was calculated by adding the antibiotic mass of the soil collected in March and manure-based compost applied to the soil (Equation (6)).

M_initial_ = M_compost_ + M_soil 1_ (6)

Finally, the degrees of dissipation (R_dissipation_), residue (R_residue_), and uptake in the soil were calculated using the initial antibiotic concentration, the antibiotic concentration that remained in the soil, and the total antibiotic concentration across different parts of the crop.

### 2.8. Statistical Analysis

Triplicate measurement values were averaged and statistical analysis was performed using the Statistical Package for the Social Sciences, version 26.0 (IBM Corporation, Armonk, NY, USA). One-way ANOVA and post hoc Duncan’s tests (*p* < 0.05) were used to compare each sample’s physicochemical properties and antibiotic concentration.

## 3. Results and Discussion

### 3.1. Chemical Properties of Compost and Soil

The chemical properties of the manure-based compost are presented in Table 2. For the three manure-based compost samples, the range of mean pH values was 8.71–9.90, showing alkaline characteristics. The ranges of mean electrical conductivity (EC) values and organic matter contents (OM) were 39.0–70.5 dS/m and 78.2–90.3%, respectively. A previous study reported that the alkaline property and high values of EC and OM contents are common for typical manure-based compost [27]. Generally, manure-based compost showed neutral or alkaline properties due to it containing ammonium nitrate. Additionally, high EC values (up to 80 dS/m) and OM contents (up to 93%) can be observed because of the high salt content and mixed organic bedding material [27]. 

The mean pH and EC values of manure-based compost in the sampling site were significantly different and the OM content at sampling site 1 was much higher than those at sampling sites 2 and 3. As we did not examine the manure source, organic bedding materials, and the composting process of each manure-based compost in this study, there was a limitation to reveal the exact reason for the difference in chemical properties in manure-based compost. However, previous studies showed that the chemical properties of manure-based compost can be changed depending on different livestock species, varied organic bedding materials, and the composting process [27,28].

The chemical properties of soil collected in March and September are shown in Table 3. Soil texture analysis showed that the soil was sandy loam in sampling sites 1 and 2, whereas soil in sampling site 3 was classified as sandy clay loam. The mean pH and EC values and OM content in soil collected in March were 5.10–6.25, 0.24–0.38 dS/m, and 1.91–2.65%, respectively. After cultivation, the pH, EC, and OM contents of soil collected in September were increased in all three sampling sites. The highest increase was observed in soil pH (28.9%) and EC (154.1%) in sampling site 2, and in OM (58.1%) in sampling site 1. Previous studies reported that applying manure-based compost can increase soil pH and EC because of the increased CO_2_ and nutrient concentration [28,29,30]. When manure-based compost or organic amendments (biochar) were applied to soil, the concentration of CO_2_ was increased because of enhancing microbial activity [31]. In addition, nutrient solubility increased as the soil pH increased [32,33]. Although we did not examine the microbial activity in this study, we presumed that an increase in microbial activity can contribute to an increase in soil pH and EC in the study plot. 

### 3.2. Concentrations of Antibiotics in Manure-Based Compost and Soil

The concentrations of six VAs detected in manure-based compost are shown in Table 4. In manure-based compost, all VAs were detected except for SMX. The highest concentration of all five detected VAs was observed at sampling site 3, and significantly different concentrations of VAs were observed across the three sampling sites. Among them, the highest mean concentration of CTC (234.19 μg/kg) was observed at sampling site 3, and the lowest mean concentration of SMZ (3.52 μg/kg) was measured at sampling site 1. 

The concentrations of VAs in manure-based compost are highly dependent on the amount of antibiotics administrated to different livestock [29,30]. Zhang et al. [34] reported that the average concentrations of CTC, OTC, TC, and SMZ in the refined commercial compost made from livestock manure were 29.8 μg/kg, 138.6 μg/kg, 55.3 μg/kg, and 15.2 μg/kg, respectively. However, much higher mean concentrations of CTC (3585 μg/kg) and SMZ (956 μg/kg) were detected in chicken-manure-based compost in Zhejiang province, China [35]. Another study also measured concentrations of CTC and OTC in swine, chicken, and cow manure and reported that the average concentration of CTC was in the order: cow (2.22 mg/kg) > swine (1.15 mg/kg) > chicken (1.09 mg/kg), and swine (2.69 mg/kg) > chicken (1.55 mg/kg) > cow (1.24 mg/kg) for OTC [36]. In addition, biological and abiotic factors such as microbial activity and thermal processes can affect the degradation rates of VAs remaining in livestock manure during the composting process [37,38,39]. 

Concentrations of VAs in soil are summarized in Table 5. In the soil samples, none of SMZ, SMX, or STZ were quantified and only CTC, OTC, and TC were detected with the mean concentration range of 2.54–13.08 μg/kg in March and 0.52–7.87 μg/kg in September. The concentrations of CTC, OTC, and TC in soil collected in March were significantly different between sampling sites, and the highest concentration of CTC, OTC, and TC was observed at sampling site 3. A similar trend was observed for the detected VAs in soil collected in September, showing that significantly higher mean concentrations of CTC, OTC, and TC were observed at sampling site 3 compared to sampling sites 1 and 2. 

We assumed that relatively high concentrations of VAs detected in the manure-based compost at sampling site 3 (Table 6) and initial high concentrations of CTC, OTC, and TC in soil collected in March may cause the high concentrations of CTC, OTC, and TC at sampling site 3 in September. Residuals of CTC, OTC, and TC in manure-based compost from the previous year could have remained in the soil and the addition of manure-based compost containing a high concentration of CTC, OTC, and TC may increase the concentrations of those VAs in soil. Previous studies also reported that the application of manure-based compost containing high concentrations of tetracyclines increased residuals of tetracyclines in the soil [40,41]. However, more detailed information such as analyzing the control sample (crop cultivation without application of manure-based compost) and degradation rate of manure-based compost after applying in soil would be necessary to reveal the main reason for high concentrations of CTC, OTC, and TC at sampling site 3 after cultivation.

Meanwhile, none of the SMZ, SMX, and STZ were detected in soil, although SMZ and STZ were contained in manure-based compost. This result agrees with a previous study that tetracycline antibiotics were detected at a concentration of 11.69–21.46 μg/kg, whereas much lower concentrations of sulfonamide antibiotics were detected at the level of 0.01–1.22 μg/kg in soil applying livestock-manure-based compost [42]. In addition, Hamscher et al. [43] reported that the concentration of sulfonamide antibiotics in the liquid manure was higher than that of tetracycline antibiotics, but no sulfonamide antibiotics were found in soils continuously applied with liquid manure. Sulfonamide antibiotics are highly mobile and showed low adsorption capacity in soil when the soil pH was at a range of 5.5–7.0. Sulfonamide antibiotics can be in nonionized forms under weak acidic conditions in soil, and negatively charged characteristics are also produced when the soil pH is close to the pK_a2_ value (7.5), causing a desorption of sulfonamide antibiotics from the soil [44]. 

### 3.3. Concentrations of Bioaccumulated Antibiotics in Crops

The concentrations of antibiotics in crops divided into three different parts are summarized in Table 6. Among the six antibiotics, four antibiotics (CTC, OTC, TC, and STZ) were detected in a mean concentration range of 1.05–39.57 μg/kg and none of the SMZ and SMX were detected in all three crops. Among different crop species and parts, the edible part of perilla had the highest mean concentration of CTC, OTC, TC, and STZ, whereas the only antibiotic detected in maize was STZ (1.05–1.88 μg/kg) in three different parts. In soybean, OTC (9.90–12.72 μg/kg) and STZ (3.07–3.72 μg/kg) were detected in all three parts. Comparing different parts in each crop, the mean concentration levels were ordered: edible part > stem > root for all detected antibiotics.

The bioaccumulation of antibiotics in crops has been reported by many researchers [4,45,46,47,48]. Concentrations of antibiotic residuals in crop tissue can vary depending on different antibiotics and the crop species. Dolliver et al. (2007) reported the uptake of sulfamethazine at a concentration range from 0.1 to 1.2 mg/kg in corn, lettuce, and potato after the application of manure, and concluded that leafy crops have more of a tendency to accumulate antibiotics than root crops [49]. This result agrees with our study that a much higher concentration of antibiotics in perilla leaf was measured than soybean or maize. Hu et al. (2010) also measured antibiotics in organically cultivated vegetables, and reported them at a range of 0.1–532 μg/kg of VAs including tetracycline and sulfonamide antibiotics [50]. This study revealed that the uptake and accumulation of antibiotics in crops are mainly controlled by water transport and passive absorption. According to partition-limited model results, the main uptake mechanism of TC is passive absorption, because TCs have low octanol-water partition coefficient (K_ow_) properties. Moreover, the main uptake mechanism for SA is water transport, due to its high water solubility. 

### 3.4. The Fate of Veterinary Antibiotics Based on Mass Balance Analysis in Soil

**Figure 1:**
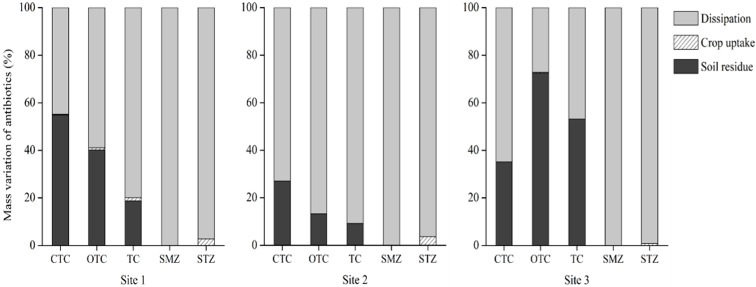
Mass balance of tetracycline and sulfonamide antibiotics in soil.

Mass balance analysis was performed to determine the fates of six VAs in soil. The relative percentages of dissipation, residue, and crop uptakes of VAs in the soil were calculated based on the mass of antibiotics in the samples (Figure 1).

Dissipation percentages of the STZ and SMZ in the three sampling sites were 83.6–97.0% and 100%, respectively, whereas CTC, OTC, and TC were 43.1–72.9%, 25.9–86.6%, and 46.1–90.8%, respectively. Antibiotics in the soil can be dissipated by various factors, such as runoff after rainfall [51] or degradation by biological and chemical processes [52,53]. Runoff and leaching during rainfall are the main dissipation routes of STZ and SMZ antibiotics into agricultural environments [54,55]. Although we did not measure antibiotics in the subsurface soil, we could assume that STZ and SMZ were leached into the sub-soil as a result of the intensive rainfall from July to September in Korea (Supplementary Data S1). Kivits et al. [56] also reported that sulfamethoxazole and sulfamethazine tend to leach into the sub-soil and can be detected in groundwater at concentrations up to 18 ng/L. 

As none of STZ, SMZ, and SMX were detected and only residues of CTC, OTC, and TC were detected in soil, the mass balance of soil residuals and crop uptake was calculated for CTC, OTC, and TC. Among them, CTC remained in the soil at rates from 27.1% to 55.4%, and OTC and TC were in the ranges from 13.4% to 73.2% and 9.2% to 53.9%, respectively. At sampling site 3, OTC and TC levels remained higher than those at sampling sites 1 and 2. However, there is a question remaining that a much higher dissipation was observed for CTC in soil at sampling site 3 after cultivation despite a higher initial concentration of CTC in soil collected in March (Table 5) and higher detected concentration of CTC in manure-based compost at sampling site 3 than at sampling sites 1 and 2 (Table 4). Assuming that the main source of detected veterinary antibiotics in soil was only manure-based compost, properties of detected veterinary antibiotics and soil chemical properties may impact the dissipation of veterinary antibiotics in soil apart from the released concentration from the source into the soil.

Previous studies have reported that the soil residuals of different VAs can vary depending on the VA properties, such as molecular structure, polarity, and degree of ionization [57,58,59]. Depending on the pK_a_ values of each VA and the soil pH, cationic, neutral, or anionic forms of VAs can be present in soil. When the pK_a_ value is low, the cationic form of VAs is present in soil and sorption is increased. In contrast, desorption is dominant when the pK_a_ value of VAs is high because the anionic form of VAs is present in the soil. Soil column experiments revealed that tetracycline antibiotics have high adsorption coefficient (K_d_) values, and remain in the soil by strongly adsorbing clay minerals and divalent cations, whereas sulfonamide antibiotics have low K_d_ values and high mobility, and can easily be transferred to surface water [60].

In addition, soil physicochemical properties (e.g., soil texture, soil pH, organic matter, and metal ion content) can affect the sorption or desorption of antibiotics in soil [61,62]. The sorption of tetracycline was increased when the soil pH was acidic and OM or clay contents were high in the soil [63]. Among those reasons affecting the sorption of tetracycline antibiotics, a high clay content can be one of the reasons for the high concentration of CTC, OTC, and TC in sampling site 3. High concentrations of clay in the soil can increase tetracycline retention, and several studies have reported higher CTC, OTC, and TC adsorption in soils with higher clay contents [64,65]. 

The average uptake percentages of CTC, OTC, TC, and STZ were 0.4%, 0.5%, 1.2%, and 2.5%, respectively. A recent study reported that only 0.1% of VAs is accumulated in plants, with 65% of VAs retained and 33% of the VAs dissipated in soil [20]. Compared with previous studies, a higher percentage of VAs was taken up by crops in this study. This difference in crop uptake percentage can be contributed by different properties of VAs and the uptake mechanism of varied crops. As mentioned in the previous section, the major uptake mechanisms of antibiotics in crops are water transport and passive absorption. In the case of sulfonamide antibiotics, water transport was preferential, whereas passive absorption was the main uptake mechanism for tetracycline antibiotics [47,50]. The uptake of VAs in soil can also vary depending on the different antibiotic classes, and the varied uptake mechanism of the crops [48]. 

In a future study, a more detailed mass balance analysis should be conducted by analyzing the control soil sample and antibiotic concentration in water samples to verify the release of VAs from the manure-based compost and to consider runoff or leaching into the groundwater. In addition, the degradation rate of released antibiotics in soil should be considered for better understanding of the fate of released VAs in soil.

## 4. Conclusions

This study evaluated the distribution and fate of six veterinary antibiotics in agricultural environments. Five of the six investigated antibiotics were detected in manure-based compost, soil, and crops. In general, the antibiotic concentrations were higher in the manure-based compost than those in soil and crops, and CTC, OTC, and TC were detected more frequently than SMX, SMZ, and STZ were in all samples. Moreover, mass balance analysis revealed the different distribution patterns and fates of each antibiotic class in the agricultural environment. In particular, CTC and OTC had higher residual characteristics in soil and crops than SMX, SMZ, and STZ did, indicating that tetracycline antibiotics can exhibit potentially adverse ecological effects. Overall, this study addressed the occurrence, fate, and distribution of VAs in soil after applications of manure-based compost for understanding the relationship of the manure-based compost–soil–crop system. Further studies should be conducted to verify the mechanisms of the sorption or desorption, dissipation, and uptake of VAs in soil.

## Figures and Tables

**Table 1:** Recovery, method detection limit, and limit of quantification of 6 veterinary antibiotics in manure-based compost, soil, and crop samples.

Veterinary Antibiotics	R^2^	Recovery (%)			Method Detection Limit (ng/kg)			Limit of Quantification (ng/kg)		
		Manure-Based Compost	Soil	Crop	Manure-Based Compost	Soil	Crop	Manure-Based Compost	Soil	Crop
CTC	0.9971	79.8	72.9	101.8	4.0	2.9	4.6	12.8	9.3	14.7
OTC	0.9992	105.8	110.0	89.9	10.4	8.7	4.0	33.0	27.6	12.8
TC	0.9982	66.3	111.9	105.9	3.3	8.2	1.5	10.5	26.3	4.7
SMZ	0.9995	108.5	81.4	80.3	2.1	2.9	1.2	6.5	9.2	3.7
SMX	0.9987	83.8	62.8	76.7	10.6	11.9	0.7	33.6	37.8	2.4
STZ	0.9993	59.1	58.8	64.4	4.5	3.6	1.1	14.3	11.3	3.2

Abbreviations of the 6 veterinary antibiotics are as follows: CTC, chlortetracycline; OTC, oxytetracycline; TC, tetracycline; SMZ, sulfamethazine; SMX, sulfamethoxazole; STZ, sulfathiazole.

**Table 2:** Chemical properties of manure-based compost in each sampling site.

Sampling Sites	pH	EC	OM
		dS/m	(%)
Site 1	9.90 ± 0.03 ^a^	39.0 ± 0.66 ^c^	90.3 ± 0.59 ^a^
Site 2	8.71 ± 0.02 ^c^	70.5 ± 0.68 ^a^	78.7 ± 6.40 ^b^
Site 3	9.68 ± 0.01 ^b^	55.4 ± 1.85 ^b^	78.2 ± 1.29 ^b^

Values of each property are mean ± standard deviation; ANOVA test was conducted and different superscript letters in the same column are significantly different at *p* < 0.05. EC and OM represent electrical conductivity and organic matter, respectively.

**Table 3:** Chemical properties of soil in each sampling site collected in March and September.

Sampling Sites	Soil Texture	Soil pH		EC dS/m		OM (%)	
		March	Spetember	March	Spetember	March	Spetember
Site 1	Sandy loam	5.10 ± 0.01 ^a^	5.30 ± 0.05 ^a^	0.24 ± 0.01 ^a^	0.52 ± 0.01 ^b^	2.10 ± 0.04 ^a^	3.32 ± 0.06 ^b^
Site 2	Sandy loam	5.43 ± 0.02 ^a^	7.00 ± 0.02 ^b^	0.37 ± 0.02 ^a^	0.94 ± 0.04 ^b^	2.65 ± 0.13 ^a^	3.47 ± 0.04 ^b^
Site 3	Sandy clay loam	6.25 ± 0.02 ^a^	7.40 ± 0.09 ^b^	0.38 ± 0.03 ^a^	0.45 ± 0.02 ^b^	1.91 ± 0.06 ^a^	2.61 ± 0.04 ^b^

Values of each property are mean ± standard deviation; ANOVA test was conducted for comparing the difference in soil properties between March and September, and different superscript letters in March and September for each soil property are significantly different at *p* < 0.05. EC and OM represent electrical conductivity and organic matter, respectively.

**Table 4:** Concentrations of veterinary antibiotics in manure-based compost.

Sampling Sites	Concentrations of Veterinary Antibiotics (Mean ± SD, μg/kg)					
	CTC	OTC	TC	SMZ	SMX	STZ
Site 1	24.38 ± 1.55 ^b^	8.06 ± 0.69 ^c^	7.41 ± 0.72 ^b^	3.52 ± 0.06 ^c^	BLD	27.94 ± 2.86 ^b^
Site 2	7.85 ± 0.52 ^c^	29.53 ± 1.30 ^b^	4.87 ± 0.14 ^c^	5.02 ± 0.72 ^b^	BLD	19.60 ± 1.85 ^c^
Site 3	234.19 ± 1.80 ^a^	38.08 ± 3.08 ^a^	44.93 ± 2.49 ^a^	28.14 ± 1.29 ^a^	BLD	187.49 ± 1.56 ^a^

BLD denotes below the limit of detection. For explanations of CTC, OTC, TC, SMZ, SMX, and STZ, see the Table 3 caption. Different superscript letters in the same column are significantly different at *p* < 0.05.

**Table 5:** Concentrations of veterinary antibiotics in soil.

Date	Sampling Sites	Concentrations of Veterinary Antibiotics (Mean ± SD, μg/kg)					
		CTC	OTC	TC	SMZ	SMX	STZ
March	Site 1	4.68 ± 0.54 ^c^	2.54 ± 0.05 ^c^	3.46 ± 0.49 ^c^	BLD	BLD	BLD
	Site 2	8.03 ± 1.28 ^b^	7.32 ± 0.31 ^b^	5.09 ± 0.72 ^b^	BLD	BLD	BLD
	Site 3	13.08 ± 0.14 ^a^	10.56 ± 0.85 ^a^	7.74 ± 0.55 ^a^	BLD	BLD	BLD
September	Site 1	2.64 ± 0.30 ^b^	1.04 ± 0.06 ^b^	0.66 ± 0.11 ^b^	BLD	BLD	BLD
	Site 2	2.18 ± 0.19 ^b^	0.99 ± 0.09 ^b^	0.52 ± 0.01 ^b^	BLD	BLD	BLD
	Site 3	5.23 ± 0.06 ^a^	7.87 ± 0.33 ^a^	4.29 ± 0.50 ^a^	BLD	BLD	BLD

BLD denotes below the limit of detection. For explanations of CTC, OTC, TC, SMZ, SMX, and STZ, see the Table 3 caption. ANOVA test was conducted separately for March and September and different superscript letters in the same column are significantly different at *p* < 0.05.

**Table 6:** Concentrations of antibiotics in the edible parts, stems, and roots of crops.

Plant Part	Crops	Antibiotic Concentrations (Mean ± SD, μg/kg)					
		CTC	OTC	TC	SMZ	SMX	STZ
Edible parts	Perilla	24.71 ± 1.51	23.60 ± 0.46	39.57 ± 1.27	BLD	BLD	4.85 ± 0.25
	Maize	BLD	BLD	BLD	BLD	BLD	1.88 ± 0.39
	Soybean	BLD	12.72 ± 0.84	BLD	BLD	BLD	3.72 ± 0.23
Stem	Perilla	9.62 ± 1.22	19.48 ± 3.23	34.50 ± 5.32	BLD	BLD	2.91 ± 0.09
	Maize	BLD	BLD	BLD	BLD	BLD	1.30 ± 0.02
	Soybean	BLD	10.91 ± 1.78	BLD	BLD	BLD	3.13 ± 0.58
Root	Perilla	4.60 ± 0.31	6.75 ± 1.10	20.79 ± 3.67	BLD	BLD	1.95 ± 0.56
	Maize	BLD	BLD	BLD	BLD	BLD	1.05 ± 0.07
	Soybean	BLD	9.90 ± 0.19	BLD	BLD	BLD	3.07 ± 0.47

BLD denotes below the limit of detection. For explanations of CTC, OTC, TC, SMZ, SMX, and STZ, see the Table 3 caption.

## Data Availability

The data presented in this study are available on request from the corresponding author.

## Supplementary Materials

The following supporting information can be downloaded at: https://www.mdpi.com/article/10.3390/toxics10050213/s1, Table S1: LC-MS/MS parameters for the analysis of antibiotics; Table S2: MRM parameters for the quantitative analysis of target antibiotics; Figure S1: Air temperature and rainfall of two provinces during cultivation period (April–October) (A) Chungnam and (B) Jenbuk province.
