# Supplementary material for: Bioaccumulation and Mass Balance Analysis of Veterinary Antibiotics in an Agricultural Environment
Source: Toxics. 2022 Apr 24;10(5):213. doi: 10.3390/toxics10050213 (PMC9147115; doi:10.3390/toxics10050213)
Supplement: Supplementary file 1 [file toxics-10-00213-s001.zip › toxics-1674114-supplementary.pdf]

toxics

Supplementary Materials: Bioaccumulation and Mass Balance Analysis of Veterinary Antibiotics in an Agricultural Environment

Jin-Wook Kim, Young-Kyu Hong, Jae-E. Yang, Oh-Kyung Kwon and Sung-Chul Kim

Table S1. LC-MS/MS parameters for the analysis of antibiotics.

Instrument	Parameter	Conditions		
HPLC	Column	Zorbax Eclipse Plus C18 3.5 μm (4.6 × 150 mm)		
	Guard Column	Security Guard cartridge Kit		
	Column temperature	25 °C		
	Flow rate	0.7 mL/min		
	Inject volume	5 μL		
	Gradient condition	Time (min)	Mobile phase A (%)	Mobile phase B (%)
		0	90	10
		2	90	10
		8	50	50
		10	100	0
		11	0	100
		11.1	90	10
		15	90	10
MS/MS	Mode	Electronic Spray Ionization		
	Polarity	Positive (+)		
	Scan type	Multiple reaction monitoring		
	Curtain gas	30 psi		
	Collision gas	High		
	Ion source temperature	500 ℃		
	Ion spray voltage	5500 V		

Mobile phase A (99.9% HPLC water + 0.1% formic acid) and mobile phase B (100% acetonitrile)

Table S2. MRM parameters for the quantitative analysis of target antibiotics.

Compound	Precursor ion (*m*/*z*)	Product ion (*m*/*z*)	DP[b] (V)	CE[c] (V)
		124.0		27
Simeton[a]	198.1	128.0	66	27
		100.1		33
		443.9		29
Chlortetracycline	479.1	462.0	1	25
		154.0		37
		426.1		25
Oxytetracycline	461.1	443.3	16	17
		365.2		41
		410.0		25
Tetracycline	445.1	153.8	51	33
		226.2		77
		186.1		23
Sulfamethazine	279.1	156.0	71	25
		124.0		29
		155.9		21
Sulfamethoxazole	254.1	108.0	1	29
		188.1		17
		155.9		19
Sulfathiazole	256.1	108.0	86	29
		193.9		46

[a]: internal standard; [b]: declustering potential; [c]: collision energy

(**A**)

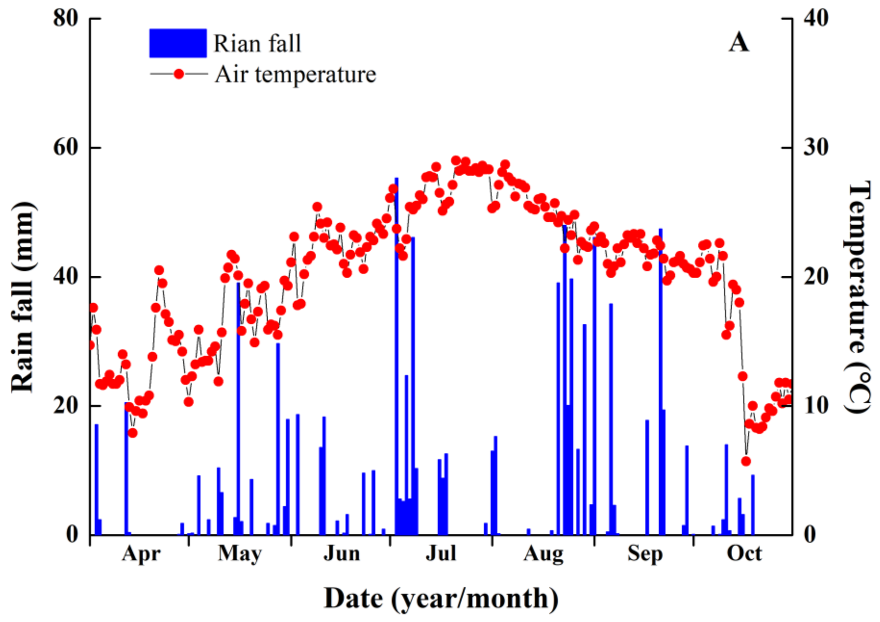

(**B**)

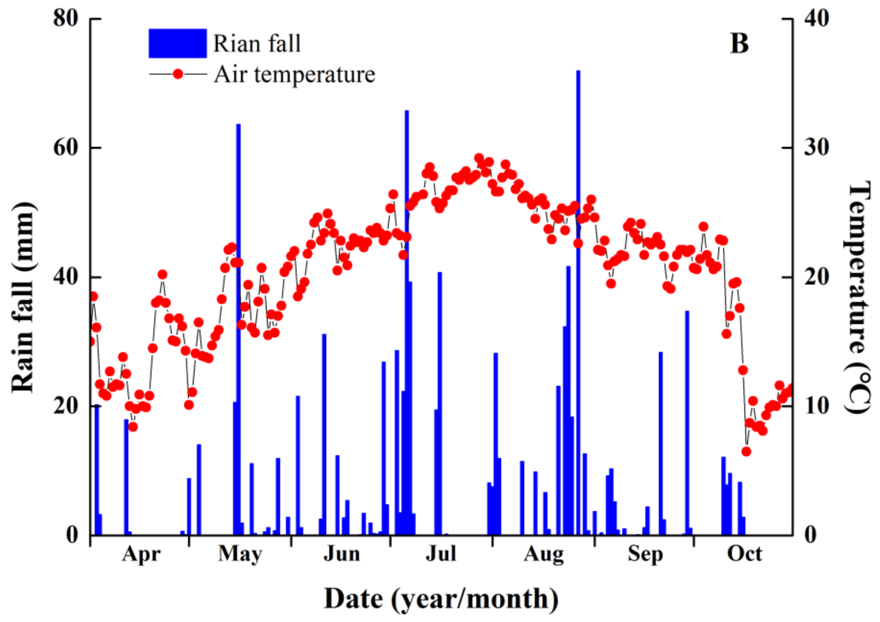

Figure S1. Air temperature and rainfall of two provinces during cultivation period (April–October) (**A**) Chungnam and (**B**) Jenbuk province.
